# Access to Liver Transplantation and Patient Survival among Asian Populations: Pre-Share 35 *vs* Post-Share 35

**Published:** 2017-11-01

**Authors:** Y. Zhang

**Affiliations:** Department of Biostatistics, School of Public Health, University of Texas Health Science Center, Houston, TX, USA

**Keywords:** Asian continental ancestry group, Health status disparities, Liver transplantation, Share 35 policy, United Network for Organ Sharing

## Abstract

**Background:**

Studies addressing ethnic disparities and trends in liver transplantation for Asian population are scant.

Objective: To examine the impact of Share 35 policy on Asian patients’ access to liver transplantation and outcomes since its implementation in June 2013.

**Methods:**

A total of 11,910 adult white and Asian patients who were registered for deceased donor liver transplantation between 2012 and 2015, was identified from the United Network for Organ Sharing database. Logistic regression and proportional hazard models with adjustment for demographic, clinical and geographic factors were used to model the access to liver transplantation and patient survival. Stratification on pre- and post-Share 35 periods was performed to compare the first 18 months of Share 35 policy to an equivalent period.

**Results:**

Comparison of the pre- and post-Share 35 periods showed a significant decrease in time on waiting list and higher proportions of patients receiving liver transplantation for Asian patients. Asians shared similar transplant rates as whites (OR: 1.15, 95% CI: 0.80–1.67) but experienced significantly longer waiting time (HR: 0.56, 95% CI: 0.34–0.92) before they received liver transplantation after Share 35 policy took effect. No significant post-transplantation survival difference was observed between Asians and whites at the 18-month outcome.

**Conclusion:**

Although benefited from the Share 35 policy, Asian patients are still at greater risk of disparities in access to liver transplantation.

## INTRODUCTION

Based on the US Census Bureau Population Projections, it is estimated that minority populations would grow by 2% per year over the next two decades. Asian population will make up approximately 11.7% of the US population by 2060 [1]. Most of the studies addressing ethnic disparities and trends in liver transplantation have focused on African-Americans and Hispanics and the information for Asian population is scant [2-5]. One preliminary study using national cancer surveillance data from 1998–2002 found that white patients were 2.56 times more likely to receive a liver transplantation than Asian and Pacific Islanders with hepatocellular carcinoma (HCC) in the pre-Model for End-Stage Liver Disease (MELD) era, but not in the post-MELD era from 2003–2005 [2]. Another study on the United Network for Organ Sharing (UNOS) database for all adult Asian liver transplantation recipients from 1998–2007 indicates that Asian ethnicity has a significant survival advantage in comparison to non-Asians [3]. 

On June 18, 2013, the Share 35 policy was implemented by the UNOS, which dramatically changed the allocation of donor livers. It mandates that regional sharing of livers for patients with a MELD score of **≥**35 is prioritized over local sharing to patients with a MELD score of <35. The intention of the policy was to reduce the waiting list mortality. Recent preliminary analyses have reported decreased mortality rates under Share 35 policy, but they did not observe any significant difference in the post-transplantation survival [6-9]. However, no study has ever investigated among Asian populations.

The objective of this study was therefore to assess the access to liver transplantation and transplantation outcomes among Asian populations under the Share 35 policy using the recent UNOS waiting list and liver database comparing the first 18 months of Share 35 policy to an equivalent period before, while accounting for geographical and other factors.

## PATIENTS AND METHODS

Data Source

UNOS is a private, non-profit organization that manages the nation’s organ transplant system under contract with the federal government [10]. Detailed descriptions of the UNOS registry have been published elsewhere [11]. Briefly, data are collected by each transplant center and transmitted to UNOS. The registry records and documents any changes in standard demographic, clinical, and laboratory information available at the time of listing, during transplantation, and post-transplantation, as well as information on the donors. The Standard Transplant Analysis and Research (STAR) file of the UNOS database contains one record per transplantation event.

The committee for the Protection of Human Subjects at the University of Texas Health Science Center at Houston approved this study.

Study Population

A total of 15,789 candidates with end-stage liver disease (ESLD), 18 years of age and older, with an initial data of registration for deceased donor liver transplantation between January 1, 2012 and March 31, 2015, was identified from the STAR wait list and liver file of the UNOS database. Only candidates with race/ethnicity defined as non-Hispanic white and Asian were selected (n=11,919). Candidates were then excluded for the following reasons: missing body mass index (BMI) (n=3), missing diagnosis (n=5), and unknown MELD score at listing (n=1). After all exclusions, a total of 11,910 patients was available for analysis. 

Study Variables

The exposure variable of primary interest was race and ethnicity as reported in UNOS records, classified as non-Hispanic white (severing as the reference group) and Asian. Other patient demographic and clinical characteristics included age at listing, sex, BMI, diagnosis, MELD score at listing, time on waiting list, waiting list outcomes, presence of HCC, presence of hepatitis C virus (HCV), presence of hepatitis B virus (HBV), organ location, and history of diabetes. 

The primary outcomes for all waiting list candidates included (1) the receipt of liver transplantation, and (2) the total time on the waiting list. Follow-up began for patients when they were initially added to the waiting list. They were then followed until the earliest of liver transplantation, death, the granting of a MELD exception score, or the end of the study. Patients received a liver transplant, alive, or lost to follow-up were censored at the date of transplantation or the last follow-up. 

Among those who received liver transplant, another primary outcome was post-transplantation patient survival. Patient survival in years was calculated from the date of liver transplantation to the date of death or the date of the last follow-up. Recipients alive or lost to follow-up were censored at the date of last follow-up.

Statistical Analysis

χ^2^ and Student’s t tests were used to compare the baseline demographics and clinical characteristics between the two racial groups as well as between pre-Share 35 and post-Share 35 periods. To incorporate the impact of geography and transplant center, marginal logistic regression models and Cox proportional hazard models were used to model the effects of Asian race on the receipt of liver transplantation, the total waiting time on the list, and post-transplantation survival. All other potential risk factors were included in the models. Stratifications between pre-Share 35 and post-Share 35 periods were also performed. All statistical analyses were performed with SAS ver 9.4 (SAS Institute Inc, Cary, NC, USA). A p value <0.05 was considered statistically significant.

## RESULTS

The baseline demographic and clinical characteristics of the 11,910 patients registered on waiting list are presented in Table 1. Compared to the pre-Share 35 cohort, Asian and white subgroups in the post-Share 35 cohort were similar in terms of age at listing, sex, HCV status, HBV status, and history of diabetes. The median time on waiting list decreased significantly from 126 days to 53.5 days for Asians and from 92 days to 53 days for whites. When it came to waiting list outcomes, both Asians (increased from 82.4% to 87.2%) and whites (increased from 81.2% to 85.2%) had higher proportions of patients receiving liver transplantation, while lower proportions of patients still waiting on the list or being too sick to receive transplantation. There were significantly more Asian patients with higher BMI, MELD score >35 registered on the waiting list, negative HCC, and more regional organs after the implementation of the Share 35 policy.

**Table 1:** Baseline demographic and clinical characteristics of waiting list candidates in the entire cohort by race: pre-Share 35 *vs* post-Share 35 periods, 2012–2015

Variable	Pre-Share 35, (n=6112)		Post-Share 35, (n=5798)			
	White, (n=5772)	Asian (n=340)	White (n=5486)	Asian (n=312)		
Mean±SD age at listing (yrs)	55.7±9.9	55.1±11.5	55.6±10.5	54.8±10.7		
Sex, n (%)								
Male	3913 (67.8)	225 (66.2)	3694 (67.3)	203 (65.1)		
Female	1859 (32.2)	115 (33.8)	1792 (32.7)	109 (34.9)		
Mean±SD BMI (kg/m^2^)	28.5±5.6	24.9±4.1	28.6±5.9	25.3±4.9		
Median (IQR) time on waiting list (d)	92 (21–246)	126 (26–350)	53 (11–146)	53.5 (7–188)		
Waiting list outcomes, n (%)					
Transplanted	4685 (81.2)	280 (82.4)	4673 (85.2)	272 (87.2)		
Still waiting	235 (4.1)	21 (6.2)	166 (3.0)	17 (5.5)		
Temporarily too sick	633 (11.0)	28 (8.2)	489 (8.9)	14 (4.5)		
Insurance issues	89 (1.5)	1 (0.3)	70 (1.3)	3 (1.0)		
Medical non-compliance	53 (0.9)	1 (0.3)	22 (0.4)	—		
Candidate withdrawn	73 (1.3)	9 (2.7)	63 (1.2)	5 (1.6)		
Candidate cannot be contacted	4 (0.1)	—	3 (0.1)	1 (0.3)		
MELD at listing, n (%)					
<35	5288 (91.6)	307 (90.3)	4739 (86.4)	244 (78.2)		
≥35	484 (8.4)	33 (9.7)	747 (13.6)	68 (21.8)		
Presence of HCC, n (%)					
No	4358 (75.5)	191 (56.2)	4340 (79.1)	208 (66.7)		
Yes	1414 (24.5)	149 (43.8)	1146 (20.9)	104 (33.3)		
Presence of HCV, n (%)					
No	3263 (56.5)	245 (72.1)	3350 (61.1)	234 (75.0)		
Yes	2344 (40.6)	87 (25.6)	1931 (35.2)	68 (21.8)		
Unknown	165 (2.9)	8 (2.4)	205 (3.7)	10 (3.2)		
Presence of HBV, n (%)					
No	4511 (78.2 )	136 (40.0)	4437 (80.9)		117 (37.5)		
Yes	935 (16.2)	186 (54.7)	801 (14.6)		182 (58.3)		
Unknown	326 (5.6)	18 (5.3)	248 (4.5)		13 (4.2)		
Organ location, n (%)					
Local	4376 (75.8)	252 (74.1)	3609 (6.8)	182 (58.3)		
Regional	1222 (21.2)	77 (22.7)	1654 (30.2)	121 (38.8)		
National	174 (3.0)	11 (3.2)	223 (4.1)	9 (2.9)		
History of diabetes, n (%)					
No	4307 (74.6)	234 (68.8)	4084 (74.4)	237 (76.0)		
Yes	1437 (24.9)	106 (31.2)	1394 (25.4)	75 (24.0)		
Unknown	28 (0.5)	—	8 (0.2)	—		

Table 2 shows the risk-adjusted odds ratios for liver transplantation rates among patients registered on waiting list for both pre- and post-Share 35 periods. Asian candidates shared similar likelihood of receiving liver transplantation as compared to white candidates, both in the pre-Share 35 era (OR: 1.04, 95% CI: 0.77–1.41) and post-Share 35 era (OR: 1.15, 95% CI: 0.80–1.67). 

**Table 2:** Logistic regression analysis results for the receipt of a liver transplant among patients registered on waiting list: pre-Share 35 *vs* post-Share 35 periods, 2012–2015. Values are ORs (95% CI) adjusted for age, sex, BMI, diagnosis, MELD score at listing, presence of HCC, presence of HCV, presence of HBV, organ location, and history of diabetes

Ethnicity	Pre-Share 35, (n=4965)	Post-Share 35, (n=5798)
White	1	1
Asian	1.04 (0.77–1.41)	1.15 (0.80–1.67)

The Cox proportional hazard regression results of the waiting time before the access to liver transplantation for both pre- and post-Share 35 periods are presented in Table 3. Asian patients had to wait approximately 50% longer on the waiting list before receiving a liver transplant compared to white patients (HR: 0.56, 95% CI: 0.34–0.92) after implementation of the Share 35 policy. 

**Table 3:** Cox proportional hazard regression analysis results for the waiting time before liver transplantation among patients registered on waiting list: pre-Share 35 *vs* post-Share 35 periods, 2012–2015. Values are HRs (95% CI) adjusted for age, sex, BMI, diagnosis, MELD score at listing, presence of HCC, presence of HCV, presence of HBV, organ location, and history of diabetes

Ethnicity	Pre-Share 35 (n = 6112)	Post-Share 35 (n = 5798)
White	1	1
Asian	0.65 (0.41–1.03)	0.56 (0.34–0.92)

We further investigated the post-transplantation survival for patients who were removed from waiting list and received a liver transplant. The Cox proportional hazard regression results are presented in Table 4. No statistically significant difference in patients’ survival between Asian and white patients was observed either in pre-Share 35 (HR: 0.82, 95% CI: 0.58–1.16) or in post-Share 35 era (HR: 0.92, 95% CI: 0.59–1.44) at the 18 month outcome.

**Table 4:** Cox proportional hazard regression analysis results of patient survival among patients who received a liver transplant: pre-Share 35 *vs* post-Share 35 periods, 2012–2015. Values are HRs (95% CI) adjusted for age, sex, BMI, diagnosis, MELD score at listing, presence of HCC, presence of HCV, presence of HBV, organ location, and history of diabetes

Ethnicity	Pre-Share 35 (n=4965)	Post-Share 35 (n=4945)
White	1	1
Asian	0.82 (0.58–1.16)	0.92 (0.59–1.44)

## DISCUSSION

Overall, the study observed significant decreased time on waiting list and higher proportion of patients receiving a liver transplant for both Asians and whites after the Share 35 policy. Although benefited from the new policy, Asian patients had similar transplantation and survival rates as their white counterparts, and still experienced 50% longer waiting time before the receipt of a liver transplant. To the best of our knowledge, this study was the first study to explore the access to liver transplantation and patient survival for Asian patients with ESLD after the Share 35 policy implemented in June 2013.

Apparently, for both Asian and white patients added to the UNOS waiting list, the Share 35 policy significantly shortened their waiting time on list, increased the percentages of regional organs, and improved their waiting list outcomes, as measured by death prior to transplantation or removal from the waiting list due to being too sick for transplantation. An important finding in this study was the lack of disparities in transplantation rates between Asians and whites after accounting for other demographic, clinical and geographic characteristics. This finding was comparable to preliminary results of recent studies after Share 35 policy took effect [4, 7-9]. Therefore, the implementation of Share 35 policy, with its emphasis on reducing mortality on waiting list, though did not lead to improved access to a liver transplant for Asian patients so far, eliminated the previously observed disparities in waiting list. However, compared to white candidates, Asians still need to wait almost 50% more before they received a liver transplant, regardless of Share 35 policy. Delayed in the access to liver transplantation was found likely to result in higher MELD scores, more advanced disease, greater disease-related morbidity, impaired access to quality pre-transplantation care, and may even be associated with worse post-transplantation outcomes [5, 12, 13]. Possible reasons behind the long waiting time included the partly shortage of eligible liver donors in the donor pool where the majority of donors are white [14], as numerous studies have already highlighted the adverse impact of donor and recipient race mismatch on post-transplantation outcomes [15, 16].

Although we studied different populations and time periods, our results were comparable to those preliminary analyses that showed there are still no significant difference in the post-transplantation patients’ survival between Asian and white patients when considering transplant center effects and other factors at 18 month outcome under the new policy. Continued investigations with longer follow-up would be necessary to assess the long-term impact of Share 35 policy, as Asian patients have long been considered to have outcomes superior to all other ethnic groups [5]. Particularly noteworthy was that the previous disparity-related or transplantation survival-related studies have only stratified on Organ Procurement and Transplant Network (OPTN) regions and some even have not correctly adjusted for geographic or transplant center factors that might affect the receipt of liver transplantation [4, 17, 18]. It has been shown that the likelihood of receiving a liver transplant varies in different parts of the country and is related to the local availability of deceased organ donors [19]. This study went beyond the previous ones that a carefully designed statistical marginal approach with a working independence assumption was incorporated so that each region or transplant center was treated as a cluster in all logistic and proportional hazard regression models to account for the impact of geographic variation and transplant center.

Moreover, an interesting finding was that the average BMI among Asian patients in this study demonstrated an increasing trend in the post-Share 35 era, though it was significantly lower than the white cohort. This is especially important for Asian populations since they had greater rates of central obesity and visceral deposition of fat and therefore, were at greater risk of metabolic syndrome and non-alcoholic fatty liver disease (NAFLD) compared to other ethnic groups with similar BMI [20, 21]. This may further contribute to increased risk of developing nonalcoholic steatohepatitis (NASH) and may result in an increasing number of patients impacting the liver transplantation waiting list [22].

This study has limitations inherent to the retrospective nature and lack of systematic data collection of the UNOS database. Racial and ethnicity data were self-reported, which was prone to misclassification bias. Second, the statistical models were limited by variables in the UNOS database. Socioeconomic factors such as education, median household income, and health insurance status were not collected; these have been considered to be conceptually linked to a patient’s choice of a transplant center as well as the access to liver transplantation [4, 23-26]. In addition, genetic variations that contributed to lower survival rates in the post-transplantation setting were not available, either. Recipients with CYP3A5 polymorphism, which occurs in 10%–40% of whites and 33% of Asians in the general population, require a higher dose of tacrolimus to achieve target trough levels for immunosuppressive therapies [27]. However, due to the size of the database, the analysis of the UNOS is so far the most possible and comprehensive analysis today.

In conclusion, this study was the first and largest to date reporting on the trends and outcomes of liver transplantation among Asian ethnicity residing in the USA under the Share 35 policy, and it adds to the existing knowledge for other ethnic groups. We would agree that the liver allocation system is getting fairer than decades age but balancing the access to the scarce medical resources remains challenge to the entire transplant community including physicians, surgeons, and policy makers. Future research studies with long-term follow-up are recommended to continuously evaluate the effectiveness of the new policy. 
